# Cell-Clearing Systems Bridging Repeat Expansion Proteotoxicity and Neuromuscular Junction Alterations in ALS and SBMA

**DOI:** 10.3390/ijms21114021

**Published:** 2020-06-04

**Authors:** Fiona Limanaqi, Carla Letizia Busceti, Francesca Biagioni, Federica Cantini, Paola Lenzi, Francesco Fornai

**Affiliations:** 1 Department of Translational Research and New Technologies in Medicine and Surgery, University of Pisa, Via Roma 55, 56126 Pisa, Italy; f.limanaqi@studenti.unipi.it (F.L.); federicacantini1@gmail.com (F.C.); paola.lenzi@unipi.it (P.L.); 2 I.R.C.C.S. Neuromed, Via Atinense, 18, 86077 Pozzilli, Italy; carla.busceti@neuromed.it (C.L.B.); francesca.biagioni@neuromed.it (F.B.)

**Keywords:** autophagy, proteasome, C9ORF72, AR, mTOR, TFEB, HSPB8, GSK3b, beta2 adrenergic receptors, nicotinic acetylcholine receptors

## Abstract

The coordinated activities of autophagy and the ubiquitin proteasome system (UPS) are key to preventing the aggregation and toxicity of misfold-prone proteins which manifest in a number of neurodegenerative disorders. These include proteins which are encoded by genes containing nucleotide repeat expansions. In the present review we focus on the overlapping role of autophagy and the UPS in repeat expansion proteotoxicity associated with chromosome 9 open reading frame 72 (*C9ORF72*) and androgen receptor (*AR*) genes, which are implicated in two motor neuron disorders, amyotrophic lateral sclerosis (ALS) and spinal-bulbar muscular atrophy (SBMA), respectively. At baseline, both *C9ORF72* and *AR* regulate autophagy, while their aberrantly-expanded isoforms may lead to a failure in both autophagy and the UPS, further promoting protein aggregation and toxicity within motor neurons and skeletal muscles. Besides proteotoxicity, autophagy and UPS alterations are also implicated in neuromuscular junction (NMJ) alterations, which occur early in both ALS and SBMA. In fact, autophagy and the UPS intermingle with endocytic/secretory pathways to regulate axonal homeostasis and neurotransmission by interacting with key proteins which operate at the NMJ, such as agrin, acetylcholine receptors (AChRs), and adrenergic beta2 receptors (B2-ARs). Thus, alterations of autophagy and the UPS configure as a common hallmark in both ALS and SBMA disease progression. The findings here discussed may contribute to disclosing overlapping molecular mechanisms which are associated with a failure in cell-clearing systems in ALS and SBMA.

## 1. Introduction

Alterations in the two major eukaryotic cell-clearing systems, autophagy and the ubiquitin-proteasome system (UPS), are promiscuously implicated in a variety of neurological disorders featuring protein misfolding, aggregation, and toxicity [1,2,3,4]. These disorders include a group in which aggregated proteins are encoded by genes containing nucleotide repeat expansions, such as Huntington disease, different forms of spinocerebellar ataxia (SCA), amyotrophic lateral sclerosis (ALS) and/or frontotemporal dementia (FTD), and X-linked spinal-bulbar muscular atrophy (SBMA, Kennedy’s disease) [5,6,7,8,9,10].

Here we focus on chromosome 9 open reading frame 72 (*C9ORF72*) and androgen receptor (*AR*) genes which are similarly affected by repeat expansions, leading to two different kinds of motor neuron disorders, namely ALS and SBMA, respectively [11,12,13,14]. Despite differing in disease frequency and clinical course, ALS and SBMA possess key overlapping features that are associated with dysfunctions of cell-clearing systems, namely protein aggregation due to expanded *C9ORF72* or *AR* within both motor neurons and skeletal muscles, as well as early neuromuscular junction (NMJ) and axonal alterations [10,15,16,17,18,19,20,21]. At baseline, both *C9ORF72* and *AR* regulate autophagy, while their aberrantly-expanded isoforms may lead to a failure in both autophagy and the UPS, further promoting protein aggregation and toxicity within motor neurons and skeletal muscles [10,21,22,23]. 

Repeat expansions within *C9ORF72* and *AR* are generally considered to produce neurotoxicity through a gain-of-function mechanism consisting of the formation of dipeptide repeat (DPR) proteins and polyglutamine-expanded AR (ARpolyQ) which aggregate in cells [10,18,24]. While being substrates for both the UPS and autophagy, these protein aggregates might in turn alter cell-clearing systems, and mostly the UPS, which is unable to process large and insoluble aggregates due to its narrow catalytic chamber [25]. Remarkably, in both ALS and SBMA, a gain-of function toxicity may concomitantly occur along with a loss of normal *C9ORF72* and *AR* function, respectively [18,19,20,26,27]. This is supposed to exacerbate the failure in cell-clearing systems, and mostly the autophagy pathway, which is physiologically activated by the normal *C9ORF72* and *AR* [22,23,28]. This is magnified in the case of *C9ORF72* repeat expansions, which often synergize with additional genetic disease modifiers to produce frank toxicity through alterations of cell-clearing systems [29]. In fact, a plethora of mutated genes which concur with *C9ORF72* repeat expansions are known to alter autophagy and the UPS [11,30,31,32]. 

It is noteworthy that regardless of the causative mechanism, disease symptoms in ALS and SBMA are bound to a failure of neurotransmission which may precede protein aggregation [33,34,35]. In particular, early alterations within the skeletal muscle, NMJ, and sensory/motor axons are emerging as a primum movens in both ALS and SBMA [33,35,36,37,38]. In line with this, both diseases are being redefined as neuromuscular synaptopathies, remarking the importance of proteostasis within the sensory–motor system as a whole [36]. In this scenario, autophagy and the UPS hold a center stage, being intermingled with each other and with endocytic/secretory pathways to regulate axonal homeostasis and neurotransmission beyond the clearance of potentially toxic protein aggregates [39,40,41,42]. As we shall see, a plethora of proteins operating at the NMJ and which are altered in ALS and SBMA, are bound to autophagy and/or UPS activities [43,44,45]. This is the case for agrin, acetylcholine receptors (AChRs), and adrenergic beta2 receptors (B2-ARs), which are implicated in NMJ maintenance [43,44,45,46]. 

In the present manuscript, after providing an overview on the promiscuous roles of autophagy and the UPS in proteoxicity within motor neurons, axons, and muscle cells in ALS and SBMA, we move to discuss potential molecular mechanisms bridging autophagy and UPS alterations with early NMJ alterations. In particular, we focus on *C9ORF72* and specific genes which may concur with *C9ORF72* repeat expansions to foster disease progression and proteotoxicity through alterations of cell-clearing systems. This is taken as a paradigm to be compared with repeat expansions similarly affecting *AR* in SBMA. The findings here discussed may contribute to disclosing overlapping molecular mechanisms in ALS and SBMA. 

## 2. Cell-Clearing Systems and *C9ORF72* Repeat Expansions in ALS

*C9ORF72* repeat expansions are currently the major genetic cause of familial ALS and/or FTD worldwide while occurring with lower frequencies in sporadic ALS and/or FTD as well [11]. In detail, GGGGCC hexanucleotide repeat expansions within the first intron of *C9ORF72* lead to *C9ORF72* haploinsufficiency and loss-of-function, and/or production of RNA foci and dipeptide repeat (DPR) proteins forming toxic aggregates in motor neurons, with the two mechanisms not being necessarily mutually exclusive [18,20,26]. Besides motor neurons, skeletal muscle cells also experience pathological changes due to the *C9ORF72* mutation. In fact, in skeletal myocytes differentiated from induced pluripotent stem cells (iPSCs) of *C9ORF72*-ALS patients, the occurrence of DPR proteins is accompanied by abnormalities in the expression of mitochondrial genes and a high susceptibility to oxidative stress, as well as increased expression and aggregation of TAR DNA-binding protein 43 (TDP-43) [47]. Supporting these findings, DPR-related pathology is detected in the skeletal muscle of ALS patients with *C9ORF72* repeat expansion [48]. 

Remarkably, both *C9ORF72* loss-of-function and production of DPR protein aggregates are bound to alterations of cell-clearing systems. *C9ORF72* acts a component of the autophagy initiation complex which is composed of ULK1-RB1CC1-ATG13-ATG101 [49]. Autophagy initiation by *C9ORF72* is mediated by a direct interaction between ATG13 and the isoform-specific carboxyl-terminal DENN and dDENN domain of *C9ORF72* [49]. Downregulation of *C9ORF72* in cell lines and primary neurons impairs autophagy by hampering Rab1a-dependent trafficking of the ULK1 autophagy initiation complex to the phagophore [22]. This in turn, promotes the accumulation of p62-positive puncta similar to what is observed in C9-ALS/FTD patient-derived neurons [22]. In primary hippocampal neurons cultured from *C9ORF72*-knockout mice, a reduction in dendritic branching and spine density occurs, which is associated with an impairment of autophagy due to reduced ULK1 levels [49]. Thus, besides protein degradation, *C9ORF72*-dependent promotion of ULK1-mediated autophagy has a key role in neuronal and dendritic morphogenesis. 

**Figure 1:**
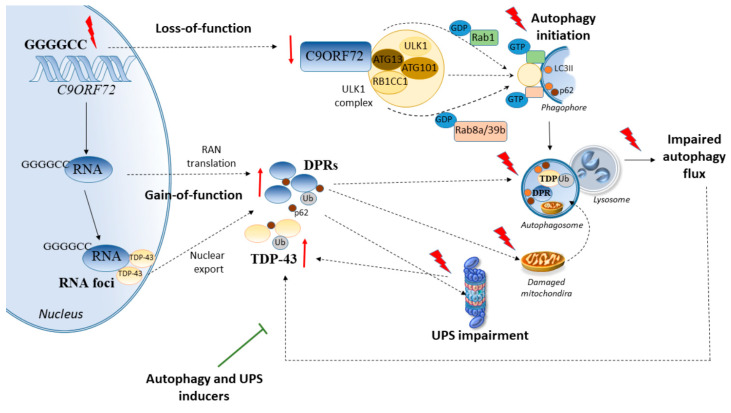
Chromosome 9 open reading frame 72 (*C9ORF72*) repeat expansions alter autophagy and the ubiquitin proteasome system (UPS) through a combination of loss- and gain-of-function mechanisms. Reduced *C9ORF72* levels due to GGGGCC expansions may synergize with dipeptide repeat (DPR)-dependent gain of toxicity through autophagy and UPS deficits. In detail, *C9ORF72* loss-of-function impairs autophagy initiation by hampering the recruitment of Rab1, Rab8/39, and ULK1 complex to the phagophore. At the same time, through a gain-of function mechanism, *C9ORF72* repeat expansions lead to the formation of DPR proteins through a repeat-associated non-AUG (RAN) translation and/or through formation of RNA foci that sequester proteins such as TAR DNA-binding protein 43 (TDP-43) which are exported from the nucleus to the cytoplasm. This leads to the formation of cytosolic protein aggregates that alter mitochondria and impair the UPS while engulfing autophagy compartments. A failure in autophagy flux along with UPS catalytic activity eventually promotes further DPR protein and TDP-43 aggregation, which can be prevented by autophagy or UPS inducers. Flashlights indicate mutations or alterations.

Recently, a disease mechanism in ALS resulting from reduced *C9ORF72* levels which can synergize with DPR-dependent gain of toxicity through autophagy and UPS deficits has been proposed [18,19]. In mice expressing a 450 repeat *C9ORF72* transgene that does not encode the *C9ORF72* protein, inactivation of the endogenous *C9ORF72* alleles exacerbates ALS phenotype and the accumulation of DPR proteins by occluding the autophagy activity [18]. This implies a double-hit pathogenic mechanism, whereby reduced expression of *C9ORF72* synergizes with an impairment of DPR clearance fostering their accumulation and toxicity in ALS [20]. This is largely bound to an autophagy impairment, which exacerbates the accumulation of DPR proteins deriving from translation of sense and antisense repeats, eventually promoting neurotoxicity. Conversely, pharmacological autophagy activators prevent these effects [20]. Data from cell lines, primary neurons, transgenic mice, and patient tissue indicate that DPR proteins derived from *C9ORF72* repeat expansion, especially poly-glycine-alanine (GA) and poly-proline-alanine (PA), impair the UPS besides autophagy [19,24,50,51,52]. Remarkably, this is associated with TDP-43 cytoplasmic mislocalization, aggregation, and toxicity, which occurs in the absence of *TARDBP* mutations [19]. In detail, cell-to-cell transmission of DPR proteins inhibits the UPS in neighboring cells, both cell-autonomously and non-cell-autonomously. In turn, UPS inhibition exacerbates the accumulation of TDP-43 which is ubiquitinated specifically within the nuclear localization signal at lysine 95 [19]. Conversely, administration of the UPS activator rolipram completely blocks DRP-dependent mislocalization and aggregation of TDP-43 [19]. In line with this, DPR proteins physically associate with proteasomes to inhibit the degradation of ubiquitinated substrates, while administration of UPS activators occludes the toxic effects of DPR on motor neuron survival (Figure 1) [24]. These findings are in line with neuropathological investigations showing that repeat expansions in *C9ORF72* are associated with ubiquitin- and p62-containing cytoplasmic inclusions, being either positive or negative for TDP-43 [11,53,54,55]. However, most of the abovementioned studies did not focus on the potential differences between the effects of UPS vs. autophagy activity and the specific type of DPR, which indeed may vary in aggregation dynamics and toxicity, while occurring within neuronal inclusions other than TDP-43-positive ones.

### 2.1. C9ORF72 Synergizes with Genetic Disease Modifiers to Alter Cell-Clearing Pathways

A stream of evidence suggests that *C9ORF72* depletion might not lead per se to major neuronal toxicity while contributing to ALS pathogenesis by directly interfering with additional genetic disease modifiers [29,56]. In fact, a number of additional mutated genes may co-occur with *C9ORF72* repeat expansions, contributing to the pleiotropic clinical and pathological phenotypes observed in ALS, including the ALS–FTD spectrum [11]. These include mutations in superoxide dismutase 1 (*SOD1*), transcription of RNA activating protein/TAR DNA-binding protein (*TARDBP*), fused in sarcoma (*FUS*), optineurin (*OPTN*), ubiquilin-2 (*UBQLN2*), progranulin (*PGRN*), ataxin-2 (*ATXN-2*), valosin-containing protein (*VCP*), and dynactin (*DCTN1*), among others [11,54,57,58,59,60,61,62,63,64]. Remarkably, mutations in these genes are per se bound to alterations of autophagy and the UPS, suggesting a possible mechanism through which they might add on the *C9ORF72* expansion-related pathophysiology. The link among cell-clearing systems dysfunctions and mutated proteins such as SOD1, TDP-43, FUS, OPTN, and UBQLN2 has been thoroughly reviewed in the literature [3,30,31,32,65], thus it will be only briefly mentioned here. In detail, misfolded SOD1, TDP-43, FUS are all substrates of the UPS and autophagy, with large and insoluble oligomer species being preferentially degraded by autophagy [66,67,68,69,70,71,72]. In turn, these protein aggregates may impair both the UPS and autophagy, while enhancing the UPS and/or autophagy prevents SOD1, TDP-43, and FUS protein aggregation and toxicity within both motor neurons and muscle cells in various ALS models [66,67,68,69,70,71,72,73,74].

UBQLNs bind the ubiquitin chains which are attached to variety of aggregation-prone proteins, fostering their delivery and degradation by either the UPS or autophagy [75,76,77]. Thus, it is not surprising that mutations in *UBQLN2* are associated with an impaired protein degradation by both the UPS and autophagy in ALS [65,78,79]. 

**Figure 2:**
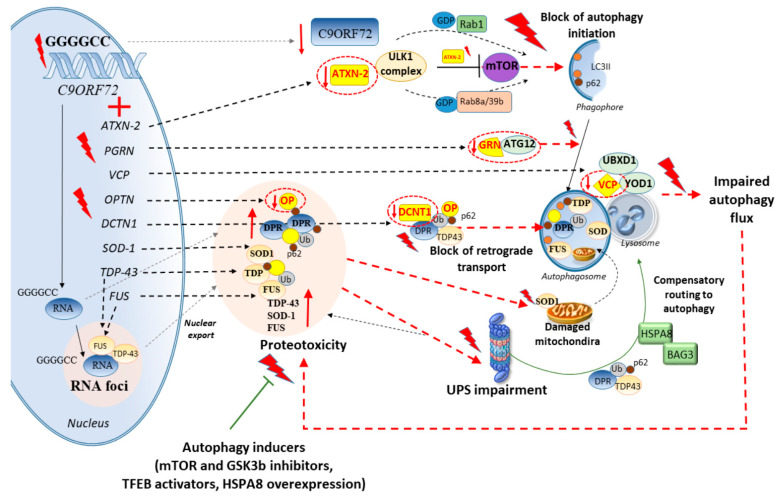
Mutated genes synergizing with *C9ORF72* repeat expansions to produce proteotoxicity through autophagy and UPS impairment. Ataxin-2 (*ATNX-2*) mutations may synergize with *C9ORF72* repeat expansions to hamper autophagy initiation through occlusion of *ATNX-2*-mediated mTOR inhibition. Progranulin (*PGRN*) mutations may hamper autophagosome formation by occluding the recruitment of ATG12. Valosin-containing protein (*VCP*) mutations may contribute to hampering autophagy flux by occluding the interaction with proteins (UBXD1 and YOD1) which mediate the fusion with lysosomes. Optineurin (*OPTN*) mutations occlude the targeting of ubiquitinated substrates or aggresomes to the UPS and autophagy pathways. Dinactin (*DCNT1*) mutations occlude the retrograde transport of ubiquitinated substrates or aggresomes to the autophagy pathway. Superoxide dismutase 1 (*SOD1*), *TDP-43*, and *FUS* mutations contribute to increase the amount of intracellular protein aggregates which impair mitochondrial homeostasis while overwhelming the UPS and autophagy. TDP-43 and FUS may also be sequestered within *C9ORF72*-induced RNA foci to be subsequently exported from the nucleus to the cytoplasm, thus potentiating the accumulation of aggregated proteins in the cytoplasm. In the context of a UPS impairment, the chaperones heat shock protein family A (Hsp70) member 8 (HSP8)/Bcl-2-associated athanogene 3 (BAG3) may be recruited as a compensatory attempt to route misfolded/aggregated proteins to the autophagy pathway. Nonetheless, a failure in autophagy flux may eventually promote protein aggregation and toxicity, which can be prevented by various autophagy inducers. Flashlights indicate mutations and alterations.

Finally, OPTN is a multifunctional autophagy receptor which possesses a ubiquitin-binding domain and it plays important roles in vesicle trafficking, maintenance of the Golgi apparatus, and autophagosome maturation. Mutations in *OPTN* may impair autophagy both through a loss-of function mechanism and through the formation of misfolded and aggregated proteins [80]. In fact, overexpression of wild type *OPTN* decreases protein inclusions which are induced by mutated *OPTN* [80]. This occurs in cooperation with the UPS through K63-linked polyubiquitin-mediated autophagy [80], which is in line with recent studies showing that OPTN may be a preferential target of the UPS [81]. This is largely bound to the recently-identified E3 ubiquitin ligase Hrd1, which increases the UPS-dependent degradation and microtubule-dependent aggresome formation of OPTN [81]. Besides these genes, less frequent mutated genes such as *ATXN-2*, *VCP*, *PGRN*, and *DCTN1* may concur with *C9ORF72* in ALS [60,61,62,63], while potentiating alterations of cell-clearing systems, as discussed below [82,83,84,85] (Figure 2). 

#### 2.1.1. ATXN-2

Depletion of *C9ORF72* in neurons mildly impairs autophagy by disrupting a molecular complex which acts as a GDP/GTP exchange factor for RAB8 and RAB39 [29,56]. This leads to the accumulation of TDP-43 and p62 aggregates, which intriguingly, is not associated with frank toxicity [29,56]. Instead, dramatic effects on cell survival are documented in neurons bearing nucleotide expansions within both *C9ORF72* and *ATXN-2*, a gene implicated in ALS besides SCA [56]. In line with this, intermediate *ATXN-2* repeat lengths are likely to make *C9ORF72* expansion carriers more susceptible to the development of motor neuron disease [61]. This is confirmed in animal models, where *C9ORF72* haploinsufficiency combined with *ATXN-2* intermediate polyglutamine repeats (30Q), markedly exacerbates ALS progression and proteotoxicity, which occurs through a powerful inhibition of the autophagy pathway [29]. When coupled with autoptic findings documenting high levels of pathologic TDP-43 in the motor cortex and spinal cord of ALS patients with *C9ORF72* and *ATXN-2* expansions [86], these pieces of evidence suggest that autophagy impairment may be a mechanistic link between TDP-43 aggregation and ALS-related repeat expansions. 

Remarkably, *ATXN-2* mutations are per se bound to an impairment of autophagy, which might explain the more severe alterations which are observed upon a combination with *C9ORF72* expansions. In fact, ataxin-2 is an intrinsically-disordered protein which acts as an autophagy inducer through inhibition of the mammalian target of rapamycin complex 1 (mTORC1) signaling [87]. In yeast, ataxin-2 binds to TORC1 specifically during respiratory growth, to inhibit TORC1 through a methionine-rich, low complexity region. This region causes phase separation and forms reversible fibrils while enabling self-association into assemblies that are required for TORC1 inhibition [87]. Mutant ataxin-2 that weakens phase separation in vitro exhibits reduced capacity to inhibit TORC1, causing consistent metabolic disturbances due to autophagy inhibition and mitochondrial dysfunctions [87]. In line with this, a CAG repeat expansion in the *ATXN-2* gene leads to mitochondrial dysfunction and autophagy inhibition, which goes along with caspase-8- and caspase-9-mediated apoptosis and production of reactive oxygen species (ROS) in vitro [82]. These events are prevented by administration of either autophagy inducers or compounds promoting oligomer dissolution [82]. This suggests that similarly to DPR proteins arising from *C9ORF72* repeat expansions, mutant *ATXN-2* may also lead to potentially toxic oligomers which can be targeted by autophagy [82]. 

#### 2.1.2. VCP

Mutations in the ubiquitously-expressed valosin-containing protein (*VCP*) gene, which occur in ALS besides inclusion body myopathy (IBM) associated with Paget’s disease of bone and FTD, lead to autophagy alterations and TDP-43-positive, ubiquitinated inclusions within both neurons and muscle cells [83]. Despite not being *VCP* mutations a major cause of ALS, pathogenic hexanucleotide expansions have been identified in the *VCP* 5’UTR of *C9ORF72*-ALS cases [62]. Since VCP is essential to autophagosome maturation both at baseline and during UPS inhibition [88], it is conceivable that a loss of VCP function might synergize with *C9ORF72* expansions to occlude autophagy-dependent degradation of ubiquitinated proteins. VCP, in cooperation with the UPS, is also key to promoting autophagy activity upon lysosomal damage. In fact, VCP moves to damaged endosomes and lysosomes where the UPS components UBXD1, PLAA, and YOD1 are concomitantly recruited to foster the removal of p62-shuttled, K63-linked ubiquitinated substrates [89]. This is key to degrading ubiquitinated substrates within damaged endosomes and lysosomes while promoting autophagosome formation. In line with this, by intermingling with endocytic and autophagy pathways, VCP promotes the degradation of TDP-43 and FUS, while TDP-43 and FUS aggregates in turn, impair VCP-dependent protein turnover [90]. As we shall see, reduced expression of VCP also occurs in SBMA models featuring mutant ARpolyQ [10], providing a possible molecular bridge among nucleotide repeat expansions, impaired protein degradation, and neuromuscular disease. 

#### 2.1.3. PGRN

A few reports showed that progranulin (*PGRN*) mutations may concur with *C9ORF72* repeat expansions [60] while acting as a disease modifier in ALS through earlier onset and shorter survival [91]. PRGN is a key regulator of autophagy and it is critically involved in motor and sensory axonal alterations which frequently occur in ALS (including *C9ORF72* patients and experimental models) and also in SBMA [26,37,92,93,94,95,96,97,98,99]. *PGRN* overexpression in sensory neurons attenuates neuropathic pain after sciatic nerve injury and accelerates nerve healing [84]. Such an effect is bound to the interactions of PGRN with the autophagy-related proteins ATG12 and ATG4b, as well as lysosomal and endocytic proteins. In line with this, defective autophagy is detected in *PGRN*-deficient neurons. This is associated with cell death, which is prevented by *PGRN* overexpression [84]. Likewise, in vivo, nerve injury produces an impairment of autophagy flux in dorsal ganglia sensory neurons and nerves, while PGRN enhances nerve healing and prevents the occurrence of protein aggregates in the injured nerves [84]. In these conditions, inhibition of the autophagy flux by hydroxychloroquine occludes the beneficial effects provided by PGRN, indicating a critical role of autophagy in the mechanisms of action of PGRN in sensory neurons and axons [84]. Similar to what is observed in sensory axons, knockdown of *PGRN* genes in zebrafish produces alterations in motor axons being characterized by short axonal outgrowth and aberrant branching [100]. Remarkably *PGRN* overexpression rescues motor axonopathy associated with either *PGRN* deficiency or TDP-43 aggregation [100], which is likely bound to autophagy activation. This is supported by evidence from zebrafish models, where the expression of *C9ORF72*-related DPR consistently induces a motor axonopathy which is rescued by the autophagy-and UPS-related protein p62 [93]. Remarkably, trehalose, an mTOR-independent activator of autophagy which exerts beneficial effects in both ALS and SBMA models [10,101,102,103], enhances *PGRN* expression in both iPSC-derived human neurons carrying a *PGRN* mutation and in the brains of *PGRN* haploinsufficient mice [104]. This suggests that autophagy inducers may be potential therapeutics for neurodegenerative diseases featuring peripheral neuropathy.

In line with these findings, autophagy alterations are now emerging as a common mechanism in peripheral neuropathies [105], which frequently occur in ALS and SBMA as well as in specific inherited disorders. Despite the increased recognition that sensory, and mostly proprioceptive neurons and fibers are affected in ALS, to date only mutated SOD1 and TDP-43 has been shown to directly affect sensory neurons and axons [96]. Nonetheless, recent studies unraveled that *C9ORF72* expansions, similar to mutated SOD1 and TDP-43, are highly toxic to axons and substantially inhibit axonal mitochondrial and vesicular transport, likely via a combination of gain- and loss-of-function mechanisms [26,106]. Since rescuing autophagy prevents the accumulation of damaged mitochondria associated with early axonal clogging in SOD1(G93A)-ALS models [94,95], it is worth investigating whether an autophagy impairment following *C9ORF72* mutations may similarly produce axonal alterations. 

#### 2.1.4. DCTN1

Axonal transport defects consistently contribute to axonal alterations and motor neuron degeneration in ALS and also in SBMA [21,94,107,108,109,110]. In fact, mutations in the genes coding for the retrograde motor complex dynein/dynactin occur in both ALS, ALS/FTD, and SBMA patients and animal models [63,64,108,109,110]. Recent studies showed that motor neuron disease-linked mutations in dynactin (*DCTN1*) may lead to both DCTN1 dysfunction and DCTN1 protein aggregation [85,111]. In line with a deleterious role of dynactin loss-of-function, the depletion of DCTN1 in mice produces a consistent loss of spinal cord motor neurons along with NMJ disintegration and muscle atrophy, which is associated with accumulating autophagosomes and lysosomes witnessing for vacuole transport defects [111]. On the other hand, consistent with a potentially toxic role of *DCTN1* mutations, overexpression of transcription factor EB (TFEB) promotes the autophagy-dependent clearance of mutant (G59S) DCTN1 aggregates while preventing cytotoxicity [85]. At baseline, the UPS is the primary degradation system for both wild type (WT) and mutated (G59S) DCTN1, while autophagy is recruited to clear mutated DCTN1 protein aggregates when the UPS is inhibited [85]. 

Similar compensatory, yet promiscuous mechanisms between autophagy and the UPS are reported in models of motor neuron disease following dynein alterations. In detail, dynein-mediated retrograde transport is key to shuttle misfolded or aggregated proteins toward the perinuclear region of the cells, where they are either degraded by autophagy or stored into the aggresome [21]. Inhibition of dynein-mediated retrograde transport is known to occlude the targeting of misfolded species to autophagy. However, in cell models of expanded polyGP-*C9ORF72*, the UPS is recruited as a compensatory response to prevent protein aggregation following inhibition of dynein-mediated retrograde transport [21]. The same effects are observed in cells expressing mutant *SOD1* and *TDP-43*. In detail, UPS recruitment and the clearance of polyGP proteins, SOD1 and TDP-43 is associated with an increase in heat shock protein family A (Hsp70) member 8 (HSPA8) cochaperone Bcl-2-associated athanogene 1 (BAG1) [21]. The latter reroutes protein cargoes to the UPS in a dynein-independent manner when autophagy-dependent protein degradation is impaired [21].

## 3. Cell-Clearing Systems and AR Nucleotide Repeat Expansions in SBMA

Analogously to what occurs in *C9ORF72*-ALS, tandem repeats in exon 1 of the *AR* gene in SBMA lead to an abnormal CAG expansion which produces a long polyglutamine tract (polyQ) in the AR protein [12]. The mutant AR (ARpolyQ) misfolds, and upon activation by the AR ligand testosterone, it forms cytoplasmic and toxic nuclear aggregates through a gain-of function mechanism. Despite neurotoxicity being largely associated with the formation of nuclear aggregates, nuclear localization of ARpolyQ is necessary though not sufficient for toxicity [112]. Remarkably, improving ARpolyQ cytoplasmic clearance contributes to decreasing ARpolyQ nuclear accumulation, which indicates that the occlusion of autophagy-dependent cytoplasmic ARpolyQ enhances the toxicity of nuclear ARpolyQ [112]. In fact, while normal AR promotes the activation of the autophagy inducer TFEB, ARpolyQ impairs TFEB-dependent autophagy flux in motor neuron-like cells [23]. The combination of the TFEB-related autophagy inducer trehalose and the antiandrogen bicalutamide which slows down AR nuclear translocation, reduces insoluble ARpolyQ within motor neurons with a higher efficiency compared with single treatments [113]. Such a combination allows an increased recognition of misfolded species by the autophagy pathway prior to their migration into the nucleus while clearing insoluble AR species which bear a very long polyQ (Q112) tract [113]. Again, in neuronal cells, the heat shock protein 90 (HSP90) inhibitor 17-(allylamino)-17-demethoxygeldanamycin (17-AAG) exerts beneficial action in SBMA [114], and this occurs through an autophagy-dependent clearance of ARpolyQ [115]. Intriguingly, 17-AAG is unable to counteract SOD1 and TDP-43 aggregation, suggesting a quite specific role for HSP90 in AR aggregation [115]. The chaperone HSPB8 also facilitates the autophagy-mediated removal of ARpolyQ aggregating species [116]. Despite not influencing p62 and LC3 levels, it does prevent p62 bodies formation while restoring autophagy flux. Trehalose, which counteracts ARpolyQ through autophagy activation, also induces *HSPB8* expression indicating the key role of HSPB8-related autophagy as a potential target against ARpolyQ toxicity [116]. 

Besides autophagy, rescuing the UPS may be key to counteracting ARpolyQ-protein degradation, as well as mitochondrial alterations and axonal transport defects in SBMA models [117]. In fact, ARpolyQ aggregates sequester mitochondria and stain positively for heat shock proteins such as HSP90 and HSP70 as well as UPS subunits, suggesting a breakdown in UPS processing [9,118]. Ubiquitin is also detected in aggregated ARpolyQ nuclear species whose proteolysis eventually requires UPS activity [119]. Recent studies showed that HSPA8 and its cochaperone BAG1 are key to rerouting ARpolyQ towards the UPS when dynein-mediated retrograde targeting of misfolded proteins to the autophagy pathway is impaired [21]. Again, in a mouse model of SBMA featuring neuronal ARpolyQ obtained through the Tet-On system, cepharanthine phytochemical from *Stephania cepharantha* decreases ARpolyQ levels both in the cytoplasm and nucleus [120]. In detail, while the UPS appears to be mostly implicated in WT AR degradation, autophagy induction following cepharanthine administration enhances the clearance of cytoplasmic ARpolyQ while providing neuroprotection [120]. 

In transgenic Drosophila, ARpolyQ produces ligand-dependent degeneration of specific neurons resulting in a rough eye phenotype, which is associated with an impairment of both the UPS and autophagy [121]. Inducing autophagy through overexpression of histone deacetylase 6 (*HDAC6*) accelerates the turnover of the ARpolyQ while lowering steady-state levels of monomeric and aggregated ARpolyQ in these fly models [121]. Treatment with the TOR inhibitor rapamycin reproduces these effects [121], suggesting a synergistic activity between autophagy and the UPS. This is in line with recent studies linking mTOR inhibition to a simultaneous activation of autophagy and the UPS [4,122,123].

### Autophagy and the UPS in SBMA Muscle and Axons

Beyond motor neurons within the spinal cord and brainstem, AR mutations also affect dorsal root ganglia neurons and skeletal muscle cells, leading to sensory dysfunctions and atrophy of bulbar, facial, and limb muscles, with CAG repeat size differentially correlating with motor- and sensory-dominant phenotypes [10,124,125,126,127]. Recent studies suggest that SBMA first manifests in skeletal muscle, prior to any motor neuron degeneration which only occurs in late-stage disease [127]. Although the polyQ expansion is known to impart a toxic gain-of-function effect upon the mutant AR protein, evidence has been provided showing that SBMA pathogenesis may concomitantly involve an AR gain-of-function toxicity and loss of normal AR function, reminiscent of what reported for *C9ORF72* repeat expansions [27]. In fact, androgens acting through the AR are important for muscle development suggesting that both loss of normal AR functions and gain of novel harmful functions can contribute to neurodegeneration and muscular atrophy [27,117]. This was addressed by crossing transgenic mice models harboring 100 *AR* glutamines (AR100) with *AR*-null mice (testicular feminization; Tfm) [27]. Absence of the endogenous AR protein in AR100Tfm mice has profound effects upon neuromuscular and endocrine features, leading to neurodegeneration and severe androgen insensitivity compared with AR100 littermates. Remarkably, AR transactivation diminishes competitively in a polyQ length-dependent way. Reduction in size and number of androgen-sensitive motor neurons in the spinal cord of AR100Tfm mice underscores the importance of normal AR action for neuronal survival and muscle function [27]. This is key since the transcriptional activity of the normal AR receptor is bound the autophagy pathway. In fact, the core autophagy genes *ATG4B*, *ATG4D*, *ULK1*, and *ULK2*, as well as *TFEB*, a master regulator of autophagosome–lysosomal biogenesis and function, are all transcriptional targets of AR [23,28]. This suggests that ARpolyQ may impair cell-clearing systems both directly, by forming large aggregates which engulf autophagy compartments and the UPS, and also indirectly, by competitively blocking AR transactivation and subsequent autophagy induction. Intriguingly, similarly to ARpolyQ which blocks AR transactivation in a length-dependent way, AR transactivation is repressed in a dose-dependent manner by glycogen synthase kinase 3beta (GSK3b) [128], a well-known autophagy modulator [73,129,130,131]. Remarkably, the suppression of AR transactivation by GSK3b is abolished by the GSK3b inhibitor and autophagy inducer lithium [128]. 

The effects of GSK3b and lithium are poorly investigated in the context of SBMA. However, lithium exerts beneficial effects in both ALS patients and experimental models, where it rescues affected motor neurons, axons, and skeletal muscles through autophagy induction and restoration of mitochondrial homeostasis [73,95,131,132,133,134,135,136,137]. Remarkably, in human muscle cultures, GSK3b activity is also enhanced by UPS inhibition, while treatment with lithium rescues UPS activity while preventing protein aggregation [138]. These findings add to the vast body of evidence linking lithium-induced inhibition of GSK3b activity with autophagy induction, showing that besides autophagy, GSK3b inhibition may empower the UPS. 

This is key since RNA-sequencing studies recently identified the UPS as one of the major pathways being disrupted in the muscle of *ARpolyQ*-knockin mice [139]. In fact, numerous UPS genes are downregulated in *AR113Q*-expressing muscle, encoding approximately 30% of UPS subunits and 20% of E2 ubiquitin-conjugating enzymes. These changes are age, hormone, and glutamine length-dependent [139]. Furthermore, the reduction in the expression of UPS genes and catalytic activity is associated with decreased levels of the UPS transcription factor NRF1 and its activator DDI2. In fact, the downregulation of *NRF1* or *ADRM1* Drosophila orthologues reproduces ARpolyQ accumulation and toxicity. These data indicate that AR113Q muscle develops progressive UPS dysfunction promoting the accumulation and toxicity of ARpolyQ protein in SBMA [139]. Conversely, enhancing UPS-dependent clearance of AR through administration of insulin-like growth factor 1 (IGF-1) reduces AR aggregation [140]. This is documented in vitro as well as in SBMA transgenic mice overexpressing a muscle-specific isoform of IGF-1, which leads to Akt-dependent AR phosphorylation and UPS-dependent AR clearance. This is associated with a reversal of behavioral and histopathological abnormalities, and reduction of both muscle and spinal cord pathology [140]. 

Supporting a role for early impairment of the UPS in SBMA muscle, an increased BAG3:BAG1 ratio along with autophagy markers is detected in the muscle of AR113Q mice, suggesting a preferential routing of misfolded proteins to the autophagy pathway [141]. Recent studies in stabilized skeletal myoblasts show that ARpolyQ forms testosterone-inducible insoluble aggregates which are processed by both the UPS and autophagy. Intriguingly, while the UPS clears both WT AR and ARpolyQ, autophagy clears mostly ARpolyQ and it is early activated by ARpolyQ itself [10]. Nonetheless, ARpolyQ, even in the absence of testosterone, reduces the expression of two autophagy-related proteins BAG3 and VCP, eventually impairing autophagy response in ARpolyQ s-myoblasts [10]. Overexpression of BAG3 ameliorates ARpolyQ clearance, while the treatment with trehalose induces complete ARpolyQ degradation, suggesting that ARpolyQ may eventually impair autophagy besides the UPS in muscle cells [10]. 

**Figure 3:**
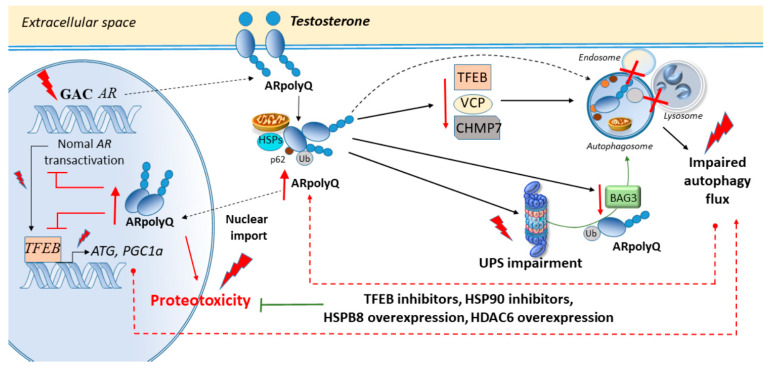
Androgen receptor (*AR*) repeat expansions produce proteotoxicity through autophagy and UPS impairment. A CAG expansion in the *AR* produces a long polyglutamine tract (polyQ) in the AR protein. The mutant AR (ARpolyQ) misfolds, and upon activation by the AR ligand testosterone, it forms cytoplasmic and toxic nuclear aggregates through a gain-of function mechanism. ARpolyQ occludes autophagy by decreasing transcription factor EB (TFEB), VCP, charged multivesicular body protein 7 (CHMP7), and also BAG3, which is activated as a compensatory response following UPS impairment by ARpolyQ. ARpolyQ nuclear accumulation may also lead to a loss of normal AR transactivation while further impairing autophagy. In fact, while normal AR promotes the activation of the autophagy inducer TFEB, ARpolyQ impairs both normal AR transactivation and TFEB-dependent autophagy, eventually promoting the accumulation of cytoplasmic ARpolyQ species which are imported in the nucleus to produce toxicity. Boosting ARpolyQ cytoplasmic clearance through autophagy and UPS inducers contributes to decreasing ARpolyQ nuclear accumulation and toxicity.

Similar to what was observed for dynein/dynactin-related alterations in ALS, mutations in *DNCT1* in a transgenic mouse model of SBMA lead to late-onset, slowly progressive muscle weakness along with deficits in axonal caliber and NMJ integrity, indicating a distal degeneration of motor neurons [142]. Remarkably, this is associated with accumulation of enlarged lysosomes and lipofuscin granules, witnessing for an impaired fusion with autophagosomes [142]. Similar defects in autophagy flux are observed within the motor neurons and hindlimb muscle of SBMA mice models, as well as in SBMA mice embryonic motor neurons and in a human cell model of motor neuron precursor cells derived from reprogrammed patient fibroblasts [16]. In detail, ARpolyQ expansion results in early transcriptional downregulation of the charged multivesicular body protein 7 (*CHMP7*), which leads to impaired autophagy flux and alterations in the endosome–lysosome pathways. This is intriguing since CHMP7 is part of the endosomal sorting complexes required for transport (ESCRT)-III complex that also includes CHMP2, which is mutated in ALS [143]. These proteins sort ubiquitinated proteins from endosomes to the lysosome through formation of multivesicular bodies while delivering autophagosomes to lysosomes [16]. Besides leading to lysosomes accumulation and impaired autophagy flux, ARpolyQ-induced *CHMP7* downregulation is also bound to gene pathways which are associated with mitochondrial clearance and axonal branching as well as NMJ formation and maintenance [16]. Among these genes, an enrichment in the GSK3b signaling pathway, which is known to promote autophagy and UPS inhibition, was identified in SBMA motor neurons along with a concomitant downregulation in pathways being involved in NMJ development [16]. Thus, autophagy and UPS alterations may play a key role in the development of SBMA by affecting early NMJ integrity while promoting protein aggregation and toxicity (Figure 3). In light of these considerations, potential mechanisms through which autophagy and the UPS may affect NMJ integrity will be discussed in the following section.

## 4. Potential Mechanisms Linking Cell-Clearing Systems and Neuromuscular Junction Alterations in ALS and SBMA

### 4.1. Autophagy and the UPS Regulate NMJ Development and Function

In Drosophila, autophagy promotes NMJ growth by reducing the levels of a mutated E3 ubiquitin ligase (highwire), suggesting that autophagy may compensate for UPS dysfunction during NMJ development [144]. Autophagy is also key for presynaptic assembly and for axon outgrowth dynamics. In fact, as shown in *Caenorhabditis elegans*, autophagosome biogenesis occurs in the axon near synapses, and this is largely bound to the presence of the integral membrane autophagy protein ATG9 [145]. In turn, alterations of autophagy in either motor neurons or skeletal muscles promote early NMJ disruption and axon degeneration [146]. This is documented in murine models bearing a transmembrane protein 184B (*TMEM184b*) mutation, which leads to early sensory–motor alterations that are associated with accumulation of stagnant, rimmed autophagy vacuoles and inclusions [146]. Remarkably, these are reminiscent of those caused by mutations in the autophagy-regulating *VCP* [146,147]. Again, the ultrastructural terminals of mutant *TMEM184b* mice feature alterations which resemble those occurring in models of neuro-axonal dystrophy caused by *PLA2G6* phospholipase mutations [146,148,149]. Finally, dystrophic presynaptic swellings which are consistently found in *TMEM184b* mutants are reminiscent of those occurring in mice bearing a spontaneous mutation in the deubiquitinating protease USP14 [146,150]. This is associated with an abnormal accumulation of UPS substrates in axon terminals [150]. These findings strengthen the evidence that autophagy and the UPS are critically involved in sensory–motor terminal structure and function. This may be relevant for early axon degeneration which occurs in neuromuscular disorders including ALS and SBMA. 

### 4.2. Autophagy and the UPS Regulate Neurotransmission at the NMJ

Besides clearing potentially toxic protein aggregates to maintain synaptic and axonal proteostasis, autophagy and the UPS play a key role in neurotransmitter release [39,40,41,42,151,152,153,154,155]. At the Drosophila NMJ, the presynaptically-enriched chaperone Hsc70-4, which is known to form a multimeric complex with HSPB8/BAG3, promotes autophagy to modulate neurotransmitter release through the turnover of specific synaptic proteins such as Unc-13, EndophilinA, WASp, and Comt/NSF [40]. Loss of autophagy slows down neurotransmission, while potentiating autophagy increases neurotransmission through the formation of a larger, readily releasable synaptic vesicle pool. Such a process is modulated by Sgt, a cochaperone of Hsc70-4, which is able to switch the activity of Hsc70-4 from autophagy-promoting toward a protein-folding activity. Thus, Hsc70-4 controls rejuvenation of the synaptic protein pool in a dual way, either by refolding proteins together with Sgt, or by targeting them for autophagy-dependent degradation [40]. Similar findings are reported in mice featuring UPS dysfunctions due to *USP14* mutations [42]. This is associated with a reduction in the size of the readily-releasable vesicle pool within the NMJ, which cannot keep pace with physiological rates of transmitter release [42]. Likewise, in mice carrying a spontaneous mutation in the E3 ubiquitin ligase *HERC1*, a reduction of the motor end-plate area is detected along with inefficient neuromuscular activity and impaired evoked neurotransmitter release at the NMJ [155]. Similarly to autophagy, the UPS at the Drosophila NMJ controls synaptic vesicle priming and neurotransmitter release probability through the turnover of UNC-13 presynaptic protein [156], which is altered in neurological disorders including ALS [131,137]. Therefore, autophagy and UPS-dependent targeting of synaptic proteins is key to orchestrating neurotransmitter release and the size of synaptic vesicles pools at the NMJ. 

### 4.3. Autophagy and the UPS Regulate nAChR Turnover at the NMJ

At vertebrate motor endplates, the conversion of nerve impulses into muscle contraction is initiated by binding of acetylcholine to its nicotinic receptor (nAChR) at the postsynapse [44]. Efficiency and safety of this process largely depend on proper localization of the receptors, which in turn, depends on autophagy- and UPS-dependent turnover. In fact, by intermingling with endocytic trafficking pathways, autophagy and the UPS orchestrate the delivery and clustering of nAChR to the postsynaptic membrane, as well as its endocytic retrieval, leading to either recycling or degradation of nAChR [44,157,158]. In detail, autophagy regulates the turnover of the endocytosed nAChR in cooperation with the E3 ubiquitin ligase, TRIM63, and SQSTM1/p62 [157]. Inhibition of the UPS in cell lines leads to a marked upregulation nAChR, which is reproduced, though at a lesser extent, by the administration of autophagy inhibitors [159]. In detail, nAChR ubiquitination and UPS-dependent degradation modulates its distribution between specialized intracellular compartments and the plasma membrane. This effect is achieved by controlling the stability of the alpha3 subunit and, consequently, the number of receptors at the cell surface [160]. Mice with defective autophagy due to skeletal muscle-specific loss of *ATG7*, display alterations of nAChR turnover and of endosome trafficking, which goes along with fragmentation of NMJs, and early synaptic dysfunction including partial denervation [15]. In line with this, nAChR expression is increased under muscle wasting conditions such as immobilization and denervation, highlighting the key role of nAChR turnover by autophagy and the UPS in these conditions [157,159]. 

Muscle denervation in ALS has a deep impact on AChR composition and distribution, and missense variants in nAChR genes are detected in sALS patients [161]. In detail, mutations within alpha3 and alpha4 nAChRs subunits lead to altered receptor trafficking associated with reduced receptor desensitization and sustained intracellular Ca(2+) concentration compared with WT-nAChR [161]. Alterations of nAChR are also reported in SBMA. Recently, a genome-wide transcriptome analysis in SBMA-derived iPSCs differentiated into spinal motor neurons revealed the involvement of synapse alterations associated with aberrant AChR clustering and NMJ formation [38]. This goes along with the upregulation of synaptic proteins which are substrates of autophagy and the UPS, such as synaptotagmin and synaptophysin [38]. Remarkably, nAChR mRNA levels are also upregulated in SBMA mice models featuring muscle AR toxicity [35,162]. This is associated with slowed synaptic potentials and reduced size of the readily releasable synaptic vesicle pool and probability of release [35], which are known to be orchestrated by the UPS and autophagy. 

Autophagy and the UPS are also bound to key proteins belonging to a complex-signaling cascade which is required for synapse formation at the mammalian NMJ [41,43]. This is the case of agrin, a motor neuron-derived proteoglycan that stabilizes the junction, and muscle-specific receptor tyrosine kinase (MuSK), a key organizer of post-synaptic components. At the level of muscle fibers, agrin normally inhibits the dispersal of AChRs while its alterations lead to endplate fragmentation [163]. In mice featuring an mTOR-dependent genetic suppression of autophagy, a downregulation of agrin occurs, which is associated with marked NMJ malformations, aberrantly distributed nAChRs, and varicose presynaptic nerve terminals in the muscle [43]. Remarkably, administration of the mTOR inhibitor torin2 reverses both autophagy and agrin downregulation in muscle and nerves while preventing NMJ alterations. Thus, autophagy failure is bound to an aberrant distribution of AChRs and NMJ malformations associated with agrin signaling alterations. Again, the UPS component PDZRN3, a PDZ domain containing the Ring ubiquitin ligase, acts as a MuSK-binding partner. PDZRN3 is concentrated at postsynapses and it promotes MuSK ubiquitination while suppressing the agrin-induced AChRs clustering. The lack of Ring domain abolishes such an effect, suggesting that the UPS is important for AChRs clustering and NMJ development [164].

### 4.4. Autophagy Converging with the Sympathetic Innervation of NMJs

**Figure 4:**
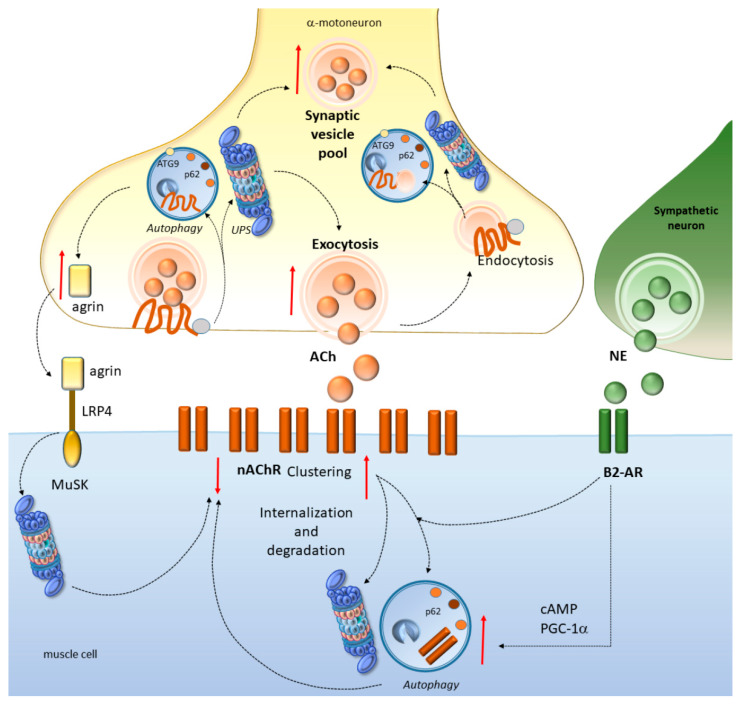
Autophagy and the UPS at the neuromuscular junction. Autophagy and the UPS promote neurotransmitter (acetylcholine, ACh) release and contribute to rejuvenating the synaptic vesicle pool through the turnover of specific proteins that are implicated in synaptic vesicle exocytosis and endocytosis. Autophagy activity is also bound the to the production of agrin, which together with muscle-specific receptor tyrosine kinase (MuSK), a UPS substrate, forms a complex that is key to maintaining the neuromuscular junction (NMJ) and to regulating nAChR at the postsynapse. Here, autophagy and the UPS are key to promote the internalization and degradation of AChR, which is also bound to the activity of adrenergic beta2 receptors (B2-ARs). In fact, B2-ARs contribute to regulating nAChR clustering and NMJ functions, likely through stimulation of autophagy via cAMP-peroxisome proliferator-activated receptor γ-coactivator 1α (PGC-1α).

Besides the tuning of autophagy and of the agrin pathway, the beneficial effects of the sympathetic innervation of NMJs are being widely investigated in the treatment of muscle wasting disorders, including ALS and SBMA [45]. In detail, sympathetic neurons make close contacts with NMJs and they form a network involving blood vessels, motor neurons, and muscle fibers, which is crucial for synapse maintenance and function [165]. Direct stimulation of sympathetic neurons leads to activation of muscle postsynaptic β2-adrenoreceptor (B2-AR) and subsequent cAMP production, which is also key to control the abundance and distribution of AChRs in NMJs [165]. These events are molecularly associated with the import of the transcriptional coactivator peroxisome proliferator-activated receptor γ-coactivator 1α (PPARGC1A, or PGC-1α) into myonuclei [165]. Remarkably, PPARGC1A is known to upregulate autophagy through a SQSTM1-dependent mechanism [166]. In turn, the UPS mediates PPARGC1A nuclear degradation and governs its cellular localization, half-life, and potential biological actions [167]. These findings provide a potential mechanistic link between sympathetic innervation and cell-clearing systems in NMJ homeostasis. This is further supported by studies showing that trehalose, which has beneficial effects in both ALS and SBMA, induces autophagy in a TFEB-dependent manner through upregulation of *PPARGC1A* as well as well-known autophagy-related genes such as *BECN1*, *LC3*, *ATG10*, *ATG12*, and *SQSTM1/p62* [101]. This is key since norepinephrine was shown to activate autophagy through B2-ARs [46,168,169], even though contradictory results have been provided documenting an activation of the mTOR pathway following chronic B2-AR administration, which is supposed to inhibit autophagy [170]. However, the fact that PPARGC1A activation similarly occurs following B2-AR stimulation and TFEB activation suggests that B2-ARs likely induce autophagy through mTOR-independent mechanisms, which calls for confirmatory studies. Altogether, these findings suggest that cell-clearing systems are key to grant NMJ homeostasis at baseline (Figure 4), while their alterations might promote NMJ derangements which occur early in ALS and SBMA.

## 5. Conclusions and Future Directions

The findings here discussed suggest that boosting autophagy/UPS activity might counteract early NMJ alterations while facilitating the removal of potentially-toxic protein inclusions, including DPR and ARpolyQ which arise from *C9ORF72* and *AR* repeat expansions, respectively. Obviously, besides ALS and SBMA, this may apply to a very broad range of neurodegenerative proteoinopathies, including those featuring poly-Q protein aggregation such as HD and SCA, where mechanisms underlying autophagy–UPS crosstalk would similarly deserve to be dealt with. Another issue that deserves further attention is the potentially-different role of autophagy vs. the UPS in DPR degradation. In fact, DPR proteins possess peculiar structures and dynamics compared with classical misfolded proteins and their toxicity may also differ for each specific DPR [171,172]. Albeit being less frequent than poly-GP and poly-GA DRP, arginine-containing DPR poly-GR and poly-PR are generally associated with greatest toxicity [172]. Again, DPR inclusions generally sequester SQSTM1/p62, though the preferred pathway of degradation is not uniform for the five DPR proteins [171]. Of all five DPR, only the polyGP seems to be efficiently removed via the UPS, while the others are apparently to be mainly degraded via autophagy. An exception is represented by polyPR, which is not significantly affected by autophagy inhibition through 3-MA administration or HSPB8 depletion [171]. This is likely due to the fact that polyPR inclusions are mostly detectable in the nucleus, where they cannot be cleared by autophagy. Remarkably, HSPB8 overexpression significantly and robustly counteracts the accumulation of insoluble species of all five DPR proteins, which suggests that the action of this chaperone may take place before polyPR nuclear import and aggregation [171]. How HSPB8 recognizes and facilitates clearance of DPR proteins remains to be determined. A peculiar feature of arginine-containing DPR proteins is their potential for post-translational modification by arginine methyl-transferases, which produces methylarginine DPR [172]. Supporting a possible contribution of methylarginine post-translational modification to poly-GR toxicity, an association between dimethylarginine-poly-GR and neurodegeneration was documented in C9-ALS and/or FTD patients [172]. A role for arginine methylation has also been documented in the pathogenesis of experimental FUS-related ALS [173]. Intriguingly, posttranslational arginine methylation is key for modulating degradation efficiency in selective autophagy by regulating the association of the cargo-receptor complex with the scaffold protein [174]. This calls for further studies investigating the potential relationship between alterations in autophagy/UPS and methyl-transferases in the mechanisms of proteotoxicity. 

## Abbreviations

UPS	ubiquitin proteasome system
C9ORF72	chromosome 9 open reading frame 72
AR	androgen receptor
ALS	amyotrophic lateral sclerosis
SMBA	spinal bulbar muscular atrophy
FTD	frontotemporal dementia
NMJ	neuromuscular junction
ARpolyQ	polyglutamine-expanded AR
DPR	dipeptide repeat
AChRs	acetylcholine receptors
B2-ARs	adrenergic beta2 receptors
TDP-43	TAR DNA-binding protein 43
SOD1	superoxide dismutase 1
TARDBP	transcription of RNA activating protein/TAR DNA-binding protein
FUS	fused in sarcoma
OPTN	optineurin
UBQLN2	ubiquilin-2
PGRN	progranulin
ATXN-2	ataxin-2
VCP	valosin-containing protein
DCTN1	dynactin
mTORC1	mammalian target of rapamycin complex 1
SQSTM1/p62	sequestosome 1/p62
HSPA8	heat shock protein family A (Hsp70) member 8
Hsc70	heat shock cognate 71 kDa protein
BAG1/3	Bcl-2-associated athanogene 1/3
TFEB	transcription factor EB
GSK3b	glycogen synthase kinase 3beta
CHMP2/7	charged multivesicular body protein 2/7
ESCRT-III	endosomal sorting complexes required for transport III
TMEM184b	transmembrane protein 184B
MuSK	muscle-specific receptor tyrosine kinase
PPARGC1A or PGC-1α	peroxisome proliferator-activated receptor γ-coactivator 1α
